# Integrating metabolomics and targeted gene expression to uncover potential biomarkers of fungal/oomycetes-associated disease susceptibility in grapevine

**DOI:** 10.1038/s41598-020-72781-2

**Published:** 2020-09-24

**Authors:** Marisa Maia, António E. N. Ferreira, Rui Nascimento, Filipa Monteiro, Francisco Traquete, Ana P. Marques, Jorge Cunha, José E. Eiras-Dias, Carlos Cordeiro, Andreia Figueiredo, Marta Sousa Silva

**Affiliations:** 1 Laboratório de FTICR e Espectrometria de Massa Estrutural, Departamento de Química e Bioquímica, Faculdade de Ciências, Universidade de Lisboa, Campo Grande, 1749-016 Lisbon, Portugal; 2 Biosystems and Integrative Sciences Institute (BioISI), Faculdade de Ciências, Universidade de Lisboa, Campo Grande, 1749-016 Lisbon, Portugal; 3 Centre for Ecology, Evolution and Environmental Changes (cE3c), Faculdade de Ciências, Universidade de Lisboa, Campo Grande, 1749-016 Lisbon, Portugal; 4 Linking Landscape, Environment, Agriculture and Food (LEAF), Instituto Superior de Agronomia (ISA), Universidade de Lisboa, 1349-017 Lisbon, Portugal; 5 Instituto Nacional de Investigação Agrária e Veterinária (INIAV), Quinta da Almoinha, 2565-191 Dois Portos, Portugal

**Keywords:** Metabolomics, Plant biotechnology, Secondary metabolism, Transcriptomics

## Abstract

*Vitis vinifera*, one of the most cultivated fruit crops, is susceptible to several diseases particularly caused by fungus and oomycete pathogens. In contrast, other *Vitis* species (American, Asian) display different degrees of tolerance/resistance to these pathogens, being widely used in breeding programs to introgress resistance traits in elite *V. vinifera* cultivars. Secondary metabolites are important players in plant defence responses. Therefore, the characterization of the metabolic profiles associated with disease resistance and susceptibility traits in grapevine is a promising approach to identify trait-related biomarkers. In this work, the leaf metabolic composition of eleven *Vitis* genotypes was analysed using an untargeted metabolomics approach. A total of 190 putative metabolites were found to discriminate resistant/partial resistant from susceptible genotypes. The biological relevance of discriminative compounds was assessed by pathway analysis. Several compounds were selected as promising biomarkers and the expression of genes coding for enzymes associated with their metabolic pathways was analysed. Reference genes for these grapevine genotypes were established for normalisation of candidate gene expression. The leucoanthocyanidin reductase 2 gene (*LAR2*) presented a significant increase of expression in susceptible genotypes, in accordance with catechin accumulation in this analysis group. Up to our knowledge this is the first time that metabolic constitutive biomarkers are proposed, opening new insights into plant selection on breeding programs.

## Introduction

Grapevine (*Vitis vinifera* L.) is one of the most cultivated fruit plants in the world, with an important economic impact in wine and table grape industries. Of the 80 known and globally distributed *Vitis* species^1,2^, *Vitis vinifera* L. is the mostly used in viticulture. As a result of its easy cultivation, vineyards longevity and numerous applications, in 2018, the global surface area for grapevine production was 7.4 Mha^1^. Grapevine cultivation requires preventive applications of agrochemicals to control several diseases, such as downy mildew [*Plasmopara viticola* (Berk. & Curt.) Berl. & de Toni) Beri, et de Toni], powdery mildew [(*Erysiphe necator* syn. *Uncinula necator* (Schweinf.) Burrill), gray mold (*Botrytis cinerea* Pers.) and black rot (*Guignardia bidwellii* (Ellis) Viala & Ravaz), that affect all the green parts of the plant and grapes^3^. However, some chemical products are not entirely efficient, with major pathogen outbreaks being reported^4,5^. Others are more efficient but have highly economic and environmental costs, besides the detrimental effects to human and animal health^6,7^. Over the last decade, there has been an increasing demand for environmentally friendly agricultural practices. With the general recommendations of the European agricultural policy encouraging the reduction of pesticides towards environmental sustainability and consumer health improvement, alternatives are needed. One possible approach is the creation of more resistant grapevine varieties through cross-breeding programs between wild *Vitis* sp. (resistant) and *V. vinifera* (susceptible), combining resistant traits with highly desired and unique grape properties. Several crossing lines inbreed with American *Vitis* species are currently commercialized as partially resistant varieties to fungal pathogens, e.g. Regent, Calardis Blanc, Solaris^8,9^.

In breeding programs, the selection for pathogen resistance traits is only possible 2 to 3 years after plant crossing, after which the more resistant seedlings are kept^10,11^. Considering the high number of newly developed seedlings, the establishment of new and advanced selection methods that can shorten this selection time and lead to a more efficient breeding process is a much-needed requirement. Grapevine genotypes possess distinct degrees of resistance to different fungal pathogens (https://www.vivc.de/)^12^. Hence, the study of different grapevine genotypes metabolomes, without stress, will uncover the innate metabolic differences between them. The full comprehension of disease resistance or tolerance mechanisms allied with the discovery of fungal/oomycete pathogen resistance-associated biomarkers in grapevine, may allow a quick and accurate identification of the seedlings that inherited the resistant trait soon after germination.

Secondary metabolites have been proven to play an important role in grapevine defences against pathogens. Several studies have been published in pathogen infection conditions which has allowed the metabolite profiling of some grapevine-pathogen interactions through various analytical instruments. Some of these metabolites have been highlighted as possible biomarkers^13–20^. For instance, the accumulation of inositol and caffeic acid are possibly related to the innate resistance^13,20^ and hexadecanoic and the monohydroxycarboxylic acids were associated to grapevine resistance^18^. Moreover, stilbenoids were already reported as key defense compounds involved in grapevine resistance to *Plasmopara viticola*, *Erysiphe necator* and *Botrytis cinerea*^16,21^.

Metabolic biomarkers have proven their value to predict phenotypical traits before they are observed^22^. In this area, metabolomics is a powerful tool due to its ability to simultaneously characterize and quantify multiple metabolites^23–25^. Due to its extreme resolution and ultra-high mass accuracy, Fourier Transform Ion Cyclotron Resonance mass spectrometry (FT-ICR-MS) is particularly powerful for an untargeted metabolome characterization, being successfully used in the study of grapevine chemical profile^14,26–28^. Additionally, metabolomics can also be used to explore metabolic pathways, uncover key enzymes involved in the biosynthesis/catalysis of metabolites and therefore genes associated with a wide range of responses. Metabolomics together with metabolic quantitative trait loci (mQTL) mapping, are being used as tools for assisting crops’ improvement, representing a breakthrough advance for the selection of offsprings with relevant traits and identification of trait-associated metabolic biomarkers^23^. This approach has been applied to potato, rice, maize and tomato^29–33^.

The present work aimed at identifying susceptibility and resistance/tolerance biomarkers through a combined approach based on untargeted metabolite profiling and targeted gene expression analysis. Eleven field grown *Vitis* genotypes (5 *Vitis* species and 6 *Vitis vinifera*) with different resistance levels to fungal/oomycete pathogens were analysed. After an untargeted metabolomics analysis by FT-ICR-MS, the most relevant metabolites discriminating susceptible and resistant/partial resistant genotypes were mapped for pathway analysis, allowing the selection of genes coding for pathway key enzymes. A targeted gene expression analysis was performed, preceded by reference gene establishment for this sample set. One candidate was identified as a possible susceptibility-associated biomarker.

## Results

### Metabolic differentiation of susceptible and resistant/partial resistant *Vitis*

**Figure 1:**
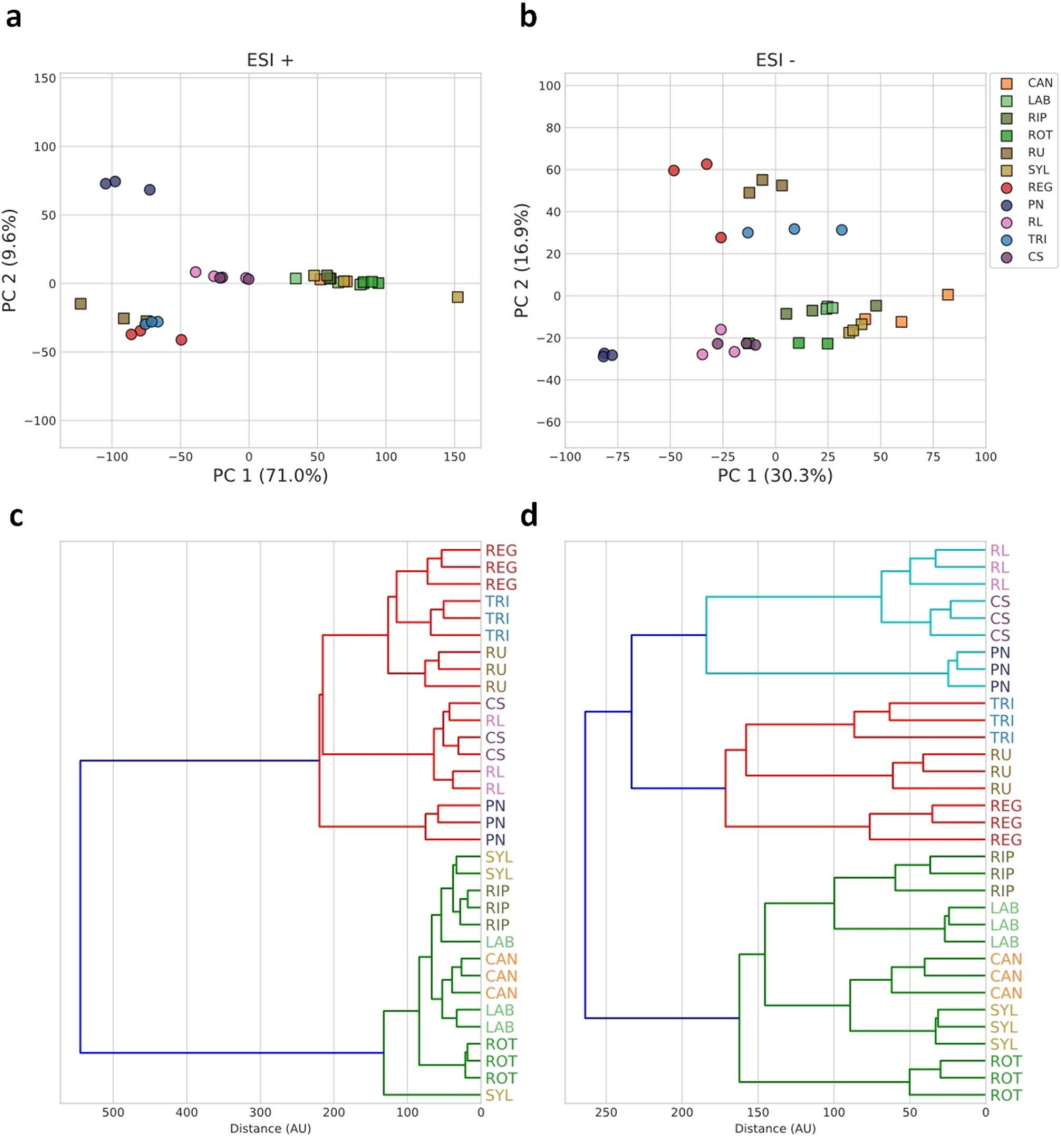
Principal component analysis (PCA) and hierarchical clustering analysis (HCA) of untargeted metabolomics obtained in positive (ESI^+^) and negative (ESI^−^) ionization modes. (**a**,**b**) PCA score plots. Squares represent wild *Vitis*, while circles represent domesticated *V. vinifera*; (**c**,**d**) HCA dendrograms. *Vitis* genotypes abbreviations are indicated in Table 1. Variance explained by each principal component is indicated in parenthesis.

**Figure 2:**
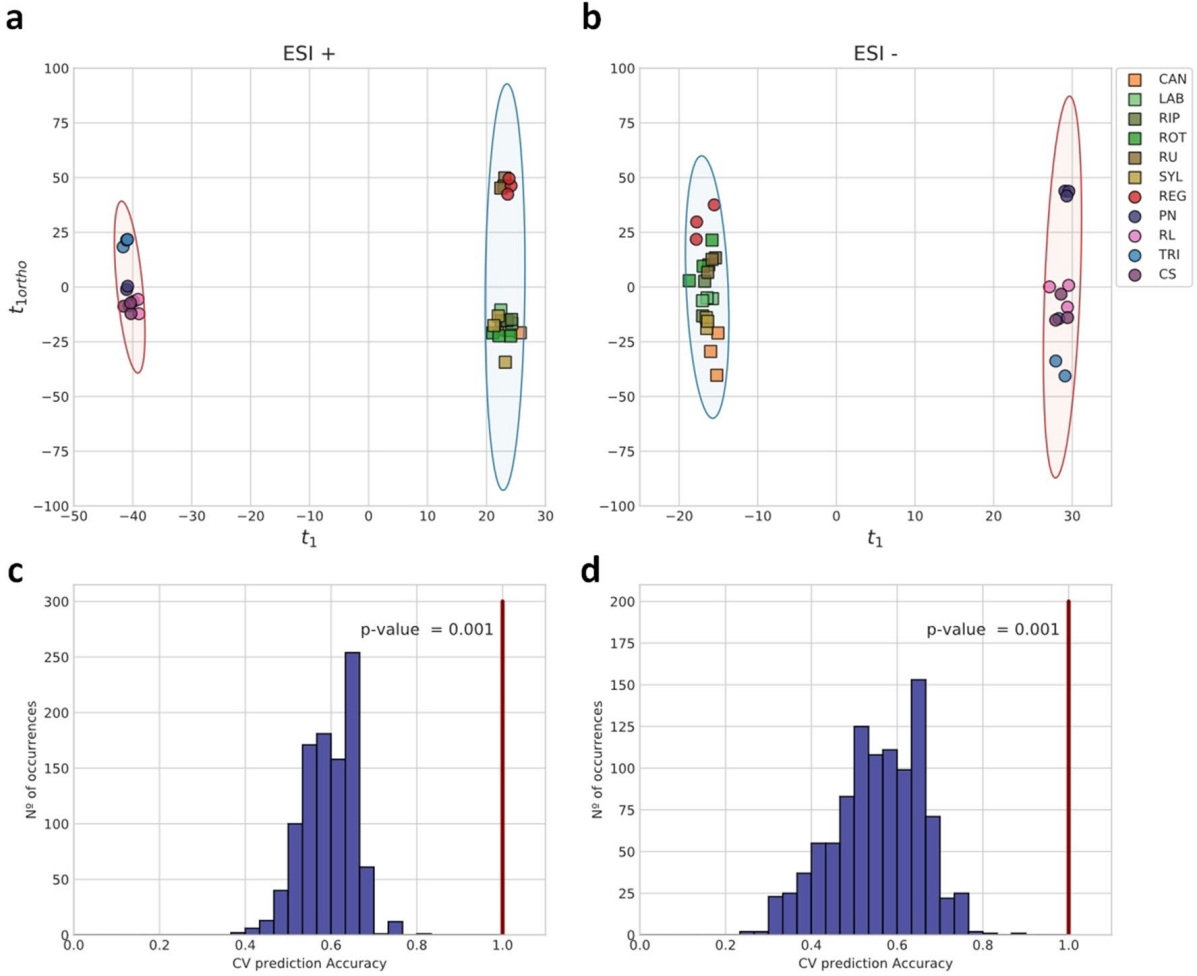
Orthogonal partial least squares discriminant analysis (PLS-DA) models for the classification into resistant/partial resistant and susceptible groups using of untargeted metabolomics data obtained in positive (ESI^+^) and negative (ESI^−^) ion modes. (**a**,**b**) Score plots for the predictive and first orthogonal components. Squares represent wild *Vitis*, while circles represent domesticated *V. vinifera*. Confidence ellipses are drawn for the two classification groups: resistant/partial resistant (blue) and susceptible (red); (**c**,**d**) Significance diagnostic showing the distribution of predictive accuracy in permutation tests and the p-value of the test for accuracy. 1000 permutations were randomly sampled. Vertical lines indicate the accuracy of model with labels non-permuted. Accuracy was estimated by sevenfold stratified cross-validation. *Vitis* genotypes abbreviations are indicated in Table 1.

Eleven *Vitis* genotypes with different tolerance to pathogens were analysed. *Vitis* species *V. labrusca*, *V. rotundifolia*, *V. riparia,* and *V. candicans* present a higher resistance/tolerance to both downy and powdery mildews and gray mold (Table 1). An untargeted metabolomics analysis using FT-ICR-MS, by direct infusion and using electrospray ionization in positive (ESI^+^) and negative (ESI^−^) ionization modes was followed. Two unsupervised approaches, principal component analysis (PCA) and hierarchical clustering, were applied to the untargeted metabolomics data to verify the analytical reproducibility and to infer inter-genotype metabolic profile similarities among the various *Vitis* samples (Fig. 1). Data reproducibility, as seen from the clustering of replicates together, was very high, an indicating that metabolome profiling of *Vitis* leaves appears to be sufficiently sensitive to distinguish the different species and cultivars. Also, in both ionization modes, a trend of separation between wild *Vitis* and *V. vinifera* cultivars can be observed in the PCA score plots (Fig. 1a,b). The dendrograms resulting from hierarchical clustering confirm this trend, since two major clusters were formed with these two sample groups (Fig. 1c,d). The only exception to this overall trend is *V. rupestris*. It is also apparent that the metabolome profile variation among *V. vinifera* cultivars seems to be larger than variation among wild species’ samples (Fig. 1a,b). The general trend of separation suggests that the multivariate metabolic phenotypes might be enough to discriminate and predict resistance or susceptibility characteristics. For that purpose, we used our metabolomics data to build classifiers for predicting the resistance or susceptibility of *Vitis* plants from their leaf metabolic profiles. We fitted Orthogonal Partial Least Squares Discriminant Analysis (OPLS-DA) models on MS intensity data using as target the inclusion on either the resistant/partial resistant group, defined by all the wild species and cultivar ‘Regent’, or the susceptible group, defined by all the remaining *V. vinifera* cultivars. Separation classifiers were built for either ESI^+^ or ESI^−^ data. Both classifiers showed very good performance. Score plots indicate that the predictor component was able to discriminate between the two groups (Fig. 2a,b) in both classifiers. Estimating model performance with sevenfold stratified cross-validation, the overall accuracy was grater that 0.98 and both R^2^_Y_ and Q^2^_Y_ metrics achieve top values with only a few orthogonal components (see Supplementary Figure F1a,b online). Furthermore, by assessing the significance of the models by permutation tests, the accuracy of permuted target label models, estimated by sevenfold stratified cross-validation has a distribution well below the reference accuracy of the non-permuted models which correspond, in both classifiers, to *p-*values of 0.001 (Fig. 2c,d).

**Table 1:** Wild *Vitis* species, *V. vinifera* subsp. *sylvestris* and grapevine cultivars analysed.

*Vitis* species	Subspecies (subsp.) or cultivar (cv.)	VIVC variety number	Abbreviation	Type of accession	Origin	Degree of resistance according to OIV descriptor 452			Overall response to fungi/oomycete pathogens
						*Plasmopara viticola*	*Erysiphe necator*	*Botrytis cinerea*	
*V. labrusca*	Isabella	5560	LAB	Wild species	United States of America	7	9	Unknown	PR/R
*V. rotundifolia*	Muscadinia Rotundifolia Michaux cv. Rotundifolia	13586	ROT	Wild species	United States of America	9	9	Unknown	PR/R
*V. riparia Michaux*	Riparia Gloire de Montpellier	4824	RIP	Wild species	United States of America	9	9	Unknown	PR/R
*V. candicans Engelmann*	Vitis Candicans Engelmann	13508	CAN	Wild species	United States of America	7	9	Unknown	PR/R
*V. rupestris Scheele*	Rupestris du lot	10389	RU	Wild species	United States of America	7	7	9	PR/R
*V. vinifera*	Subsp. *sylvestris*		SYL	Wild plant	Portugal	3	3	5	PR/R
	Subsp. *sativa* cv. Regent	4572	REG	Cultivated hybrid (crossing *V. vinifera* cv. Diana X cv. Chambourcin)	Germany	7	9	Unknown	PR/R
	Subsp. *sativa* cv. Riesling Weiss	10077	RL	Cultivated grapevine	Germany	3	3	1/3	S
	Subsp. *sativa* cv. Pinot Noir	9279	PN	Cultivated grapevine	France	3	3	1/3	S
	Subsp. *sativa* cv. Cabernet Sauvignon	1929	CS	Cultivated grapevine	France	1/3	1/3	5	S
	Subsp. *sativa* cv. Trincadeira	15685	TRI	Cultivated grapevine	Portugal	1/3	1/3	1/3	S

Species and cultivar names, type of accession, origin and response to fungi pathogens are indicated (information adapted from^47^ and https://www.vivc.de/). Classification of resistance: 1—very low; 3—low, 5—medium, 7—high, 9—very high or total. PR—Partial resistant; R—Resistant; S—Susceptible.

Univariate analysis based on the variable intensity changes between resistant/partial resistant and susceptible groups allowed the identification of several spectral features with both significant and large variation between the two groups. Even at a significance level of 0.01 for FDR-corrected *p-*values, we found 2535 features with |log2 FC| ≥ 1, 1796 in ESI^+^ and 739 in ESI. A search of these features in MassTRIX^34^ provided a putative identification of some of these peaks. A total of 190 unique masses with significant and large variation between our comparison groups were putatively annotated (see Supplementary Table S1 online).

**Figure 3:**
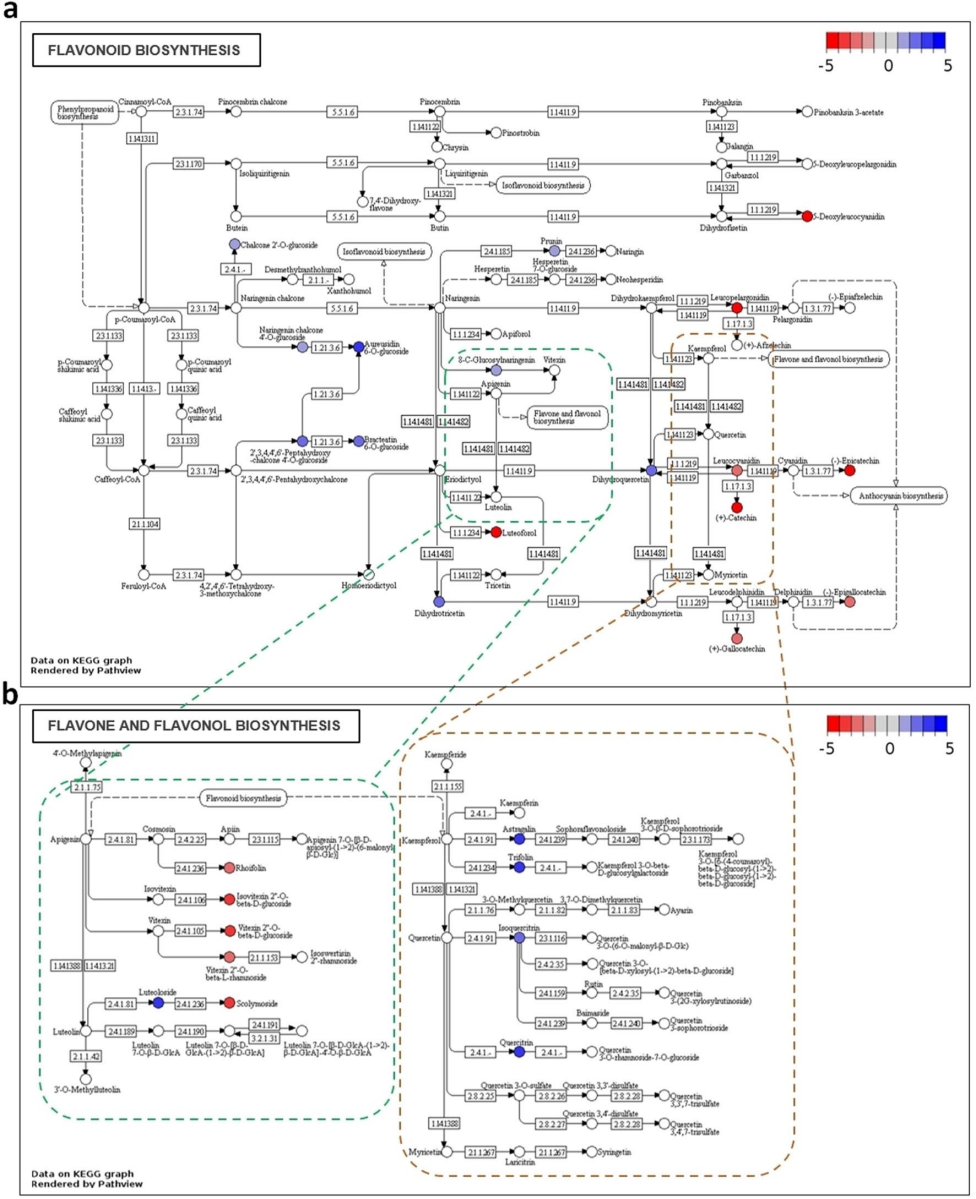
Flavonoid (**a**) and Flavone and flavonol (**b**) biosynthesis pathways from *V. vinifera* showing the discriminatory putative metabolites between resistant/partial resistant and susceptible groups (FDR corrected *p-*value < 0.01). Metabolite’s KEGG identifiers were used in the R package Pathview, coloured in agreement with their |log2(FC)| values, between resistant/partial resistant and susceptible plants: more accumulated in the resistance/tolerance group are blue, more accumulated in the susceptibility group are red and those unchanged are grey, setting the limits between − 5 and 5.

To understand the biological relevance of these discriminatory compounds in grapevine metabolism, the compounds with KEGG (Kyoto Encyclopaedia of Genes and Genomes) identifiers for database annotation were retrieved and mapped into selected pathogen defence related KEGG pathways using the R package Pathview (Fig. 3). Pathway analysis of flavonoid biosynthesis and flavone and flavonol biosynthesis, mapped 17 and 10 metabolites, respectively (Fig. 3). Among the discriminative putatively identified metabolites, we highlight catechin or epicatechin, leucocyanidin, caffeic acid, hexadecanoid acid derivatives and dodecanoic acid as more abundant in the susceptible *V. vinifera* cultivars. Quercetin 3-O-glucoside (isoquercitrin) and dihydroquercetin, together with several other flavonol 3-O-glucosides, more abundant in the resistant/partial resistant plants (see Supplementary Table S1 online).

### Reference gene selection, stability determination and expression analysis

As no reference genes were previously described for non-stressed grapevine genotypes, we selected ten candidate reference genes (RGs) based on their previous description as good qPCR control genes for *Arabidopsis thaliana*^35^ and grapevine^36–38^. Nine of the selected genes were formerly described as RGs for grapevine: 60S ribosomal protein L18 (*60S*), tetratricopeptide repeat protein 7B (*TTC7B*)], elongation factor 1-alpha (*EF1α*), ubiquitin-conjugating enzyme (*UBQ*), SAND family protein (*SAND*), glyceraldehyde-3-phosphate dehydrogenase (*GAPDH*), alpha-tubulin 3-chain (α*-TUB*), beta-tubulin 1-chain (β*-TUB*) and actin (*ACT*). Adaptor protein-2 MU-adaptin (*AP2M*) was previously described for *Arabidopsis*^35^ and sequence for its homologue in grapevine was retrieved from NCBI (https://www.ncbi.nlm.nih.gov/) (Table 2).

**Table 2:** Candidate reference genes for qPCR.

Gene (NCBI accession number)	Primer sequence	Amplicon length (bp)	Ta (°C)	Tm (°C)
*60S* (XM_002270599.3)	Fw: ATCTACCTCAAGCTCCTAGTC Rev: CAATCTTGTCCTCCTTTCCT	165	60	79.6
*TTC7B* (XM_002283371.4)	Fw: GCTCTGTTGTTGAAGATGGG Rev: GGAAGCAGTTTGTAGCATCAG	156	60	79.9
*EF1α* (XM_002284888.3)	Fw: GAACTGGGTGCTTGATAGGC Rev: ACCAAAATATCCGGAGTAAAAGA	164	60	79.7
*UBQ* (XR_002030722.1)	Fw: GCCCTGCACTTACCATCTTTAAG Rev: GAGGGTCGTCAGGATTTGGA	75	60	78.9
*SAND* (XM_002285134.3)	Fw: CAACATCCTTTACCCATTGACAGA Rev: GCATTTGATCCACTTGCAGATAAG	76	60	79.2
*GAPDH* (XM_002263109.3)	Fw: TCAAGGTCAAGGACTCTAACACC Rev: CCAACAACGAACATAGGAGCA	226	60	81.3
*ACTIN*^a^ (XM_019223591.1)	Fw: ATTCCTCACCATCATCAGCA Rev: GACCCCCTCCTACTAAAACT	89	55	77.5
*α-TUB* (XM_002285685.4)	Fw: CAGCCAGATCTTCACGAGCTT Rev: GTTCTCGCGCATTGACCATA	119	60	78.8
*AP2M* (XM_002281261.3)	Fw: CCTCTCTGGAATGCCTGATTT Rev: CTTTAGCAGGACGGGATTTA	89	55	75.0
*β-TUB* (XM_002275270.3)	Fw: TGAACCACTTGATCTCTGCGACTA Rev: CAGCTTGCGGAGGTCTGAGT	86	60	82.3

Genes, gene accession numbers, primer sequences (Fw, forward; Rev, reverse), amplicon length (bp, base pairs) and qPCR annealing (Ta) and melting (Tm) temperatures are indicated.

^a^Alternative splicing variant.

Expression stability of the candidate RGs was evaluated by three statistical algorithms, GeNorm, Normfinder and Bestkeeper, and a final rank was established with the RefFinder tool^39,40^. Ranking order of the most stable to the least stable genes is presented in Table 3. In all the *Vitis* species and *V. vinifera* cultivars analysed, genes encoding for UBQ and SAND were ranked as the most stable genes presenting the lowest M value (M = 0.859), followed by *GAPDH* (M = 0.990) and *EF1α* (M = 1.027). For all *Vitis* samples analysed, *UBQ* was considered as the most stable gene with an expression stability value (SV) of 0.552 (Table 3), followed by *AP2M* (SV = 0.744), *GAPDH* (SV = 0.745) and *β-TUB* (SV = 0.766).

**Table 3:** Candidate reference genes ranking for all *Vitis* samples calculated by GeNorm, NormFinder and BestKeeper.

Reference gene	GeNorm	NormFinder	BestKeeper		Ranking mean	Final ranking
	M value	SV	SD	*r*		
*UBQ*	0.859 (1)	0.552 (1)	1.13 (5)	0.90*	2.33	1
*SAND*	0.859 (1)	0.791 (6)	1.01 (2)	0.81*	3.00	2
*EF1α*	1.027 (3)	0.767 (5)	1.09 (4)	0.85*	4.33	3
*AP2M*	1.105 (4)	0.744 (2)	1.20 (6)	0.86*	4.33	3
*GADPH*	0.990 (2)	0.745 (3)	1.31 (8)	0.90*	4.67	4
α*-TUB*	1.181 (6)	1.122 (7)	0.92 (1)	0.59*	5.00	5
β*-TUB*	1.132 (5)	0.766 (4)	1.21 (7)	0.84*	5.67	6
*60S*	1.245 (7)	1.287 (8)	1.07 (3)	0.63*	6.33	7
*ACT*	1.439 (8)	2.056 (10)	1.63 (9)	0.69*	9.33	8
*TTC7B*	1.602 (9)	1.972 (9)	1.98 (10)	0.78*	10.00	9

Genes are ordered by the final ranking. SV, Stability value; SD, Standard deviation of Cq value; $r,$ Pearson coefficient of correlation; *p $\le$ 0.01. *p*-value associated with the Pearson coefficient of correlation; Ranking order is indicated in parenthesis.

In this study, BestKeeper analysis considered α-TUB and SAND as the most stable genes for all *Vitis* samples, with standard deviation (SD) values of 0.92 and 1.01, respectively (Table 3). 60S (SD = 1.07) was the third and EF1α (SD = 1.09) was the fourth most stable genes.

Considering the 3 algorithms, a final rank was established by RefFinder^32^. The results revealed that, in grapevine leaves, the four most stable genes for normalization were *UBQ*, *SAND*, *EF1α* and *AP2M* (Table 3).

Based on the putatively identified metabolites, respective metabolic pathways and the existing knowledge regarding markers for pathogen resistance/susceptibility in grapevine, several genes coding for enzymes in the biosynthesis or catabolism of the most discriminating metabolites were selected, namely: quercetin 3-O-glucoside (isoquercitrin), dihydroquercetin, caffeic acid, leucocyanidin, dodecanoic acid, hexadecanoic acid, catechin, epicatechin and myo-inositol.

**Figure 4:**
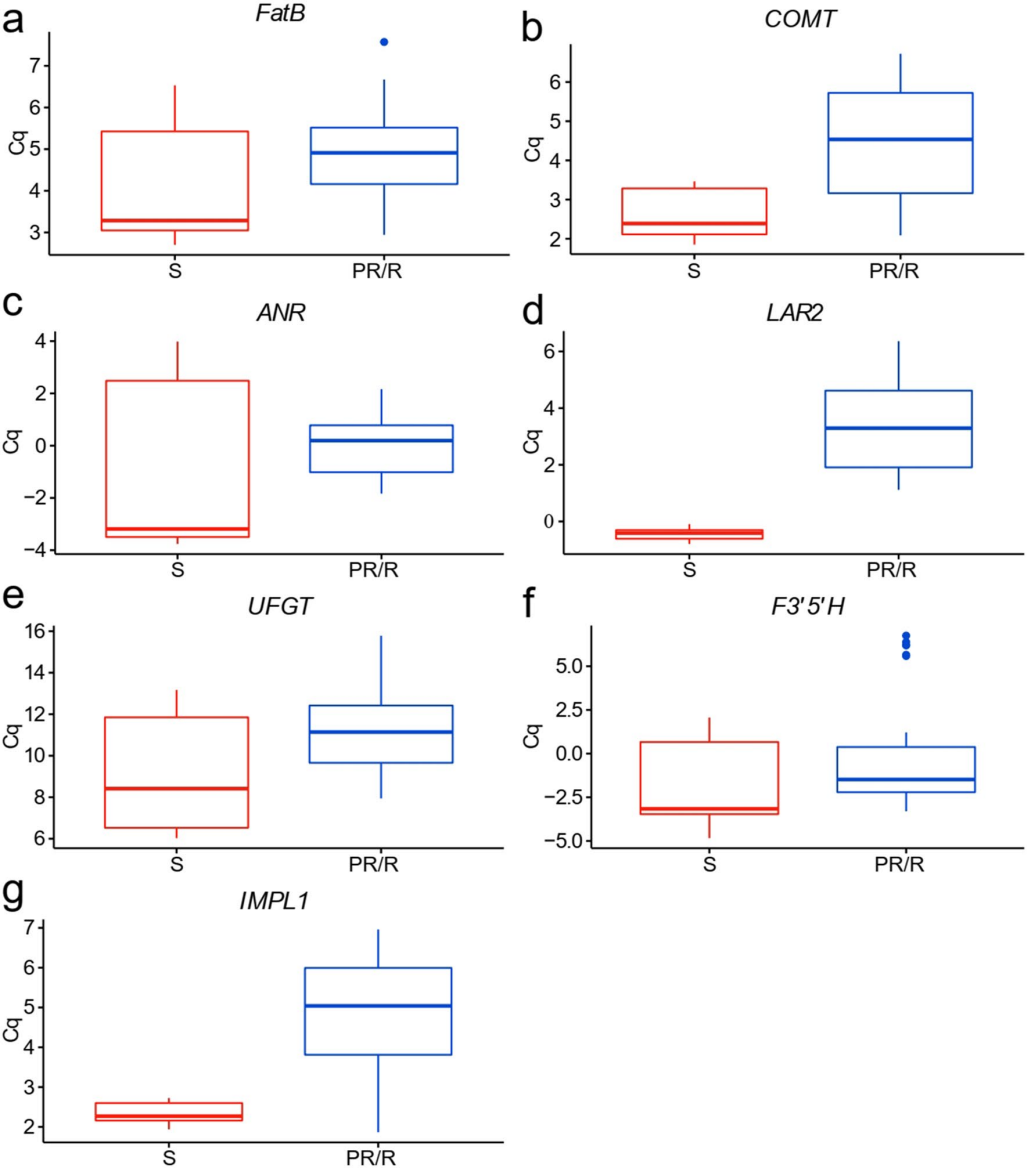
Boxplot of quantification cycles (Cq) values for the different genes of interest in susceptible (S) and partial resistant/resistant (PR/R) genotypes. (**a**) *FatB*, (**b**) *COMT*, (**c**) *ANR*, (**d**) *LAR2*, (**e**) *UFGT*, (**f**) *F3′5′H*, (**g**) *IMPL1* (gene names are indicated in Table 4). Cq values were normalized by the geometric mean of the Cq of *UBQ*, *SAND* and *EF1α*. Data for susceptible plants are represented in red and data for resistant/partial resistant are in blue.

A total of 7 genes were selected for expression analysis, coding for the following enzymes: caffeic acid O-methyltransferase (*COMT*), catalyses the conversion of caffeic acid to ferulic acid; leucoanthocyanidin reductase 2 (*LAR2*), catalyses the synthesis of catechin from leucocyanidin; anthocyanidin reductase (*ANR*), responsible for the synthesis of epicatechin from cyanidin; fatty acyl-ACP thioesterase B (*FatB*), responsible for the synthesis of hexadecanoic acid from hexadecanoyl-ACP and of dodecanoid acid from dodecanoyl-ACP; myo-inositol monophosphatase (*IMPL1*), catalyses the hydrolysis of myo-inositol phosphate into myo-inositol and phosphate; flavonoid 3′,5′-hydroxylase (*F3′5′H*), involved in several reactions in the flavonoid biosynthesis pathway; and UDP-glucose:flavonoid 3-O-glucosyltransferase (*UFGT*), catalyses the formation of flavonol 3-O-glucosides, using UDP-glucose as sugar donor (Table 4). The quantification cycle (Cq) value of the genes of interest in all *Vitis* genotypes were extracted and normalized by the geometric mean of the quantification cycles of *UBQ*, *SAND* and *EF1α*, for data normalization. For each gene, Bartlett’s test was used to access homoscedasticity of our samples and the non-parametric Wilcox–Mann–Whitney U test was performed, identifying the discriminating genes between our comparison groups. Only genes considered statistically significant in both tests (*p-*value < 0.05) were considered to be possible and reliable genetic biomarker (see Supplementary Table S2 online). *ANR, UFGT, F3′5′H and FatB* genes were, therefore, excluded (Fig. 4). On the other hand*, COMT, LAR2 and IMPL1* are clearly significantly different between susceptible and resistant/partial resistant groups, presenting lower Cq values on the susceptible groups (higher expression), (see Supplementary Table S2 online). Among these, the gene with most significance when its level is compared between groups is *LAR2* (Fig. 4, see Supplementary Table S2 online), which encodes for the enzyme leucoanthocyanidin reductase 2, responsible for the synthesis of catechin from leucocyanidin and has a higher expression in the group of susceptible plants.

**Table 4:** Genes of interest and their encoding enzymes selected for gene expression analysis.

Metabolites	Enzyme	Abbreviation	EC number	Gene NCBI accession number	Primer sequence	Amplicon length (bp)	Ta (°C)	Tm (°C)	Amplification efficiency
Caffeic acid	Caffeic acid 3-*O*-methyltransferase	COMT	EC 2.1.1.68	XM_003634113.2	Fw: GTATGACCCCAACAACTATC	88	60	78.4	1.88 ± 0.02
					Rev: GACCATGGGGAGAACTGA				
Catechin	Leucoanthocyanidin reductase 2	LAR2	EC 1.17.1.3	NM_001281160.1	Fw: TGTAACCGTGGAAGAAGATGA	92	60	80.6	1.88 ± 0.02
					Rev: ATGAAGATGTCGTGAGTGAAG				
Epicatechin	Anthocyanidin reductase	ANR	EC 1.3.1.77	NM_001280956.1	Fw: ATCAAGCCAGCAATTCAAGGA	93	60	76.2	1.88 ± 0.006
					Rev: CAGCTGCAGAGGATGTCAAA				
Dodecanoic acid/hexadecanoic acid	Palmitoyl-acyl carrier protein thioesterase B	FatB	EC 3.1.2.21/3.1.2.14	XM_019223124.1	Fw: TCGCAAACCCTAGAAACCAAT	112	60	77.5	1.93 ± 0.05
					Rev: AATGAGGGAAGGAGGAAAATG				
Myo inositol	Myo-inositol monophosphatase^a^	IMPL1	EC 3.1.3.25	XM_002276661.3	Fw: ATCCCAAACGCTACCCAAAAA	119	60	80.9	1.96 ± 0.02
					Rev: TAACAGCTTCCATCACAACCT				
Quercetin/dihydroquercetin	Flavonoid 3′,5′-hydroxylase^b^	F3′5′H	EC 1.14.14.81	XM_003632164.3	Fw: GTGGTGCCGGAGATGTTA	173	56	80.1	1.83 ± 0.05
					Rev: TGCGATGGACGGAATAAAAT				
Quercetin-3-*O*-glucoside (Isoquercitrin)	UDP-glucose:flavonoid 3-*O*-glucosyltransferase	UFGT	EC 2.4.1.91	AF000372.1	Fw: AGGGGATGGTAATGGCTGT	151	60	84.7	1.97 ± 0.01
					Rev: ATGGGTGGAGAGTGAGTTAG				

EC numbers, gene accession numbers, primer sequences (Fw, forward; Rev, reverse), amplicon length, qPCR annealing (Ta) and melting (Tm) temperatures and amplification efficiency are indicated.

^a^Alternative splicing variants.

^b^Alternative locus variants.

## Discussion

Grapevine is affected by diverse pathogens, particularly fungi and oomycetes, which, if not controlled, can affect the entire vineyard and cause a drastically reduction of the production, berry quality and yield. Downy and powdery mildews, black rot and gray mold gained the European Union’s attention and were recently flagged as the grapevine diseases with higher impact in Europe^3^. European Union is committed to “increase resilience of grape vines to pests and diseases and support the productivity of the sector in sustainable ways”, focusing on the breeding of new resistant varieties that maintain the grape qualities for wine production^3^. Its success depends on the understanding of the innate resistance mechanisms against pathogens and the identification of resistance/susceptibility-related biomarkers towards the development of assays to assist future breeding programs and introgression line analysis. The development of new crossing hybrids by the combination of wild American and Asian *Vitis*, that present innate resistance towards different pathogens^41^, with *V. vinifera* (susceptible) offer a promising alternative to the use of pesticides and contribute to an environmentally sustainable viticulture. *Vitis riparia* and *V. labrusca*, analysed in our study, exhibit resistant traits to *P. viticola*^41^ (https://www.vivc.de/) and have been effectively used in for resistance introgression. A successful hybrid example is *V. vinifera* ‘Regent’, with a broad partial resistance to the most significant pathogens (information from https://www.vivc.de/). In breeding programs, the expression of the resistant trait takes too long to be observable in the progeny. The identification of metabolic biomarkers may allow a fast and accurate identification of the seedlings that inherited the resistant traits soon after germination.

The comparison of different *Vitis* metabolomes, without being submitted to any stress, will allow the detection of relevant metabolic variations between grapevine genotypes and uncover potentially innate defence compounds that could be used as biomarkers in breeding-programs.

For that purpose, we have conducted an untargeted metabolome characterization of eleven *Vitis* genotypes presenting different levels of resistance to downy and powdery mildews and black rot. *Vitis vinifera* cultivars Pinot noir, Riesling, Trincadeira and Cabernet sauvignon are susceptible, whereas the inter-specific hybrid *V. vinifera* Regent (that combines both *Vitis vinifera* and American *Vitis* genetic background) and the *V. vinifera* subspecies *sylvestris* present a higher tolerance towards these pathogens, when compared to the other genotypes.

From our untargeted metabolomics data, two groups, *V. vinifera* cultivars and wild *Vitis*, were immediately defined and separated based on their metabolic profile. *Vitis rupestris* appears to be an exception to this overall separation trend. However, the metabolic profile of this wild *Vitis* is closer to the interspecific hybrid ‘Regent’ and cultivar ‘Trincadeira’. ‘Regent’ is considered partially resistant to downy and powdery mildews, harbouring one RPV and two REN loci^8^ (https://www.vivc.de/). On ‘Regent’ pedigree, backcrosses were made with *V. vinifera*, thus, it is expected that its metabolic profile clusters together with *V. vinifera* genotypes. Concerning the metabolome variation, it was observed to be larger in *V. vinifera* cultivars. This difference was somewhat expected, considering that domesticated grapevine cultivars present a genetic background tailored according to breeders most wanted characteristics, as the result of gene transfer during multiple crossings and selection^42,43^. For the wild species, no agronomic selection events have been pursued and thus they maintain a closer metabolic profile background.

Overall, our data predictor component was capable of discriminate between susceptible and resistant/partial resistant grapevine groups. The performance of these predictors is very encouraging in the context of sustainable agricultural practices. The prediction of resistance or susceptibility from plant leaf extracts using extreme-resolution metabolic profiling has the potential to analyse and then select crossed plants in still early development stages of their development, prior to infection, decreasing preventive pesticide use.

For the discriminant analysis, a resistant/partial resistant and susceptible group were considered, and 190 metabolites allowed the discrimination between them.

Of those, caffeic acid, catechin, epicatechin, leucocyanidin, quercetin-3-O-glucoside and derivatives, and dihydroquercetin, were found to have significant differences between the two groups. Dodecanoic acid and hexadecanoic and myo-inositol derivatives were also found to be discriminative. Some of the identified compounds were already reported as important in grapevine innate resistance^18–20^ and others as possible infection-associated resistance/partial resistance biomarkers^17,21,44–46^. In 2008, Figueiredo and co-workers, compared the metabolic profiles of a tolerant and a susceptible grapevine cultivar^20^. The accumulation of some metabolites, such as inositol and caffeic acid, was observed and a possible relation to innate resistance towards downy mildew was suggested. Also, the analysis of the leaf surface compounds from different cultivars, displaying different degrees of resistance and susceptibility to *P. viticola*, was reported by Batovska and co-workers^18^. In this study, 10 metabolites were highlighted as possible biomarkers for the prediction of downy mildew resistance and susceptibility, in which hexadecanoic acid was related to resistance in grapevine.

Some compounds were also marked as discriminatory between *Vitis* genotypes and linked to higher resistance/susceptibility to pathogens^14,15,17^. In a time-course infection assay of grapevine leaves with downy mildew, different metabolites between inoculated and control samples were identified^17^. Within these metabolites we can highlight quercetin-3-o-glucoside, myo-inositol and hexadecanoic acid, also detected in our study. Moreover, recently, Nascimento and co-workers have identified several metabolic classes, such as flavonoids, associated to grapevine defenses against downy mildew^14^. Although stilbenoids are well known plant-derived defense compounds^16,21^, no difference in resistant/partial resistant and susceptible *Vitis* genotypes was observed at the constitutive level, which is not unexpected as stilbenoids mainly occur as phytoalexins, that are produced dynamically in response to biotic or abiotic stress^21,47,48^.

Discriminative compounds between resistant/partial resistant and susceptible *Vitis* genotypes, with KEGG ID were mapped into biochemical pathways, revealing an enrichment in the flavonoid biosynthesis pathway, already described as involved in pathogen response^49,50^. These results are in line with previous studies where phenolic compounds were proven to play an important role in biotic and abiotic stress resistance^51–53^. Some of these discriminative compounds, that are end products of these pathways, were selected and genes coding for enzymes involved in their metabolic reactions were chosen, particularly from quercetin derivatives, caffeic acid, catechin/epicatechin metabolism, myo-inositol and dodecanoic acid were selected. The expression of these genes was analysed to assess their changes in resistant/partial resistant and susceptible plants.

Reference genes for our experimental conditions were defined and candidate gene expression was assessed. Three of the selected genes, *COMT*, *LAR2* and *IMPL1* allowed the discrimination between the susceptible and resistant/partial resistant groups. Albeit all these genes showed expression differences between susceptible and resistant/partial resistant *Vitis*, *LAR2* (catechin biosynthesis pathway) seems to present a higher discriminative potential. In fact, recent functional genomic studies in grapevine LAR enzymes confirmed that LAR2 is involved in the conversion of leucocyanidin into ( +)-catechin and (−)-epicatechin^54^. Moreover, catechin is a naturally occurring flavonol with high antioxidant properties. It has been previously identified as being involved in grapevine defence mechanisms^44^. Also, catechin together with other phenolic compounds, were shown to inhibit the activity of enzymes that are essential for fungal propagation and sporulation of different fungi isolated from Petri-disease-infected grapevines^46^. On the other hand, catechin can be degraded by different fungi and used as carbon source for growth^55–57^. Leaves from all susceptible *V. vinifera* cultivars had higher levels of catechin/epicatechin and an over-expression of *LAR2* gene. We hypothesize that, instead of being part of an effective defence mechanism for the plant, pathogens may be using catechin to develop and establish a successful infection.

With this work, we uncovered an important part of the metabolic map of the pathogen-resistance metabolism in grapevine, identifying key metabolic players. By assessing gene expression of key metabolic enzymes, we propose that both catechin/epicatechin and *LAR2* may be putative biomarkers of susceptibility. Despite the fact that further studies have to be conducted with a larger dataset to validate our hypothesis, we consider that our results open new insights towards the development of assays for progeny selection in breeding programs.

The study of constitutive expression and accumulation of compounds in grapevine is extremely important as it can uncovered differences associated to resistance/susceptibility to different fungal/oomycete pathogens.

## Materials and methods

### Plant material

Five wild *Vitis* species, one *Vitis vinifera* subsp. *sylvestris* (wild plants that grow into Portuguese river basins) and five *Vitis vinifera* cultivars were investigated (Table 1).

The resistance of *Vitis* genotypes was accessed through bibliographic searches following the classification of Organisation Internationale de la Vigne et du Vin (https://www.oiv.int) and the phenotype behavior observed in field conditions into the Portuguese Ampelographic *Vitis* Collection (*Colecção Ampelográfica Nacional*, CAN). CAN is property of INIAV-Estação Vitivinícola Nacional (Dois Portos), located at Quinta da Almoinha, 60 km north of Lisbon (9º 11′ 19″ W; 39º 02′ 31″ N; 75 m above sea level).

Established since 1988 and replicated to a new place in 2013 and 2014, according to maintenance conditions: established in homogeneous modern alluvial soils (lowlands) as well as well drained soil; rootstock of a unique variety (Selection Oppenheim 4–SO4) was used for all accessions including other *Vitis* species and other rootstocks represented in the field; each accession comes from one unique plant. CAN occupy nearly 2 ha of area and the climate of this region is temperate with dry and mild summer, in almost all regions of the northern mountain system Montejunto-Estrela and the regions of the west coast of Alentejo and Algarve^58^.

For plant material collection, the best possible health status was guaranteed for all accessions was confirmed: plants were tested for the principal grapevine fungal/oomycetes diseases as well as grapevine viruses (healthy genotypes and synonym accessions were planted in continuous line for didactic proposes); same trailing system (bilateral cordon, Royat), canopy maintenance and agricultural management.

Three leaves (third to fifth from the shoot to apex) were harvested in each one of 7 plants of accession (biological replicate) and immediately frozen in liquid nitrogen and stored at − 80 °C until analysis. All genotypes leaves were collected in the same day at the same time. Three biological replicates containing leaves from 2 to 3 different plants were analyzed. In overall, four wild *Vitis* species, one *Vitis vinifera* subsp. sylvestris (wild plants that grow into Portuguese river basins) and five *Vitis vinifera* cultivars were used in this experiment (Table 1).

### Metabolite extraction and FT-ICR-MS analysis

Metabolite extraction was performed as previously described^28^. Briefly, 0.1 g of plant material was extracted with 1 mL of 40% methanol (LC–MS grade, Merck)/40% chloroform (Sigma Aldrich)/20% water (v/v/v). Samples were vortexed, kept in an orbital shaker at room temperature and centrifuged for phase separation. The aqueous/methanol layer was further processed by solid-phase extraction using Merck LiChrolut RP-18 columns, pre-equilibrated and extracted with methanol. The methanol fraction was evaporated under a nitrogen stream and reconstituted in 1 mL of methanol. For FT-ICR-MS analysis, samples were diluted 1000-fold in methanol and human leucine enkephalin (Sigma Aldrich) was added for internal calibration of each mass spectrum ([M+H]^+^  = 556.276575 Da or [M−H]^−^ = 554.262022 Da). For positive ionization mode analysis (ESI^+^), formic acid (Sigma Aldrich, MS grade) was added to all samples at a final concentration of 0.1% (v/v). Samples were analysed by direct infusion on an Apex Qe 7-T Fourier Transform Ion Cyclotron Resonance Mass Spectrometer (FT-ICR-MS, Brüker Daltonics). Spectra were acquired at both positive (ESI^+^) and negative (ESI^−^) electrospray ionization modes, in the mass range of 100 to 1000 Th, with an accumulation of 250 scans for each spectrum.

### Data pre-processing and profiling by multivariate statistical analysis

Data Analysis 5.0 (Brüker Daltonics, Bremen, Germany) was used to internally calibrate each mass spectrum using leucine enkephalin for single point calibration. Peaks were considered at a minimum signal-to-noise ratio of 4. The data matrix for statistical analysis was created by peak alignment at 1 ppm difference tolerance. Only peaks occurring in more than two thirds of the replicate samples for each cultivar were selected for further analysis. Missing values were imputed by half of the global minimum value of all spectra. Data was normalized by the signal of the standard leucine enkephalin in each sample, transformed using the generalized log-transformation and Pareto scaled. The transformation with generalized log has been shown to correct for heteroscedasticity and reduce the skewness^59^. Two unsupervised methods were applied to investigate the metabolic profile similarities between *Vitis* samples. Sample Hierarchical Clustering (agglomerative) was performed, for each ionization mode, using Euclidian distance as the metric and Ward as the method for cluster aggregation. Principal Component Analysis (PCA) models for each ionization mode were also built, retaining a minimum number of principal components necessary to explain 95% of variance (12 components for ESI^+^ PCA and 15 components for ESI^−^ PCA).

Classifiers for resistant/partial resistant (n = 21) vs. susceptible (n = 12) genotypes were obtained by building Orthogonal Partial Least Squares Discriminant Analysis (OPLS-DA) models. Two target groups were defined: a “resistant/partial resistant” attributed to all the wild *Vitis* plus the domesticated *V. vinifera* ‘Regent’, and the “susceptible” group, attributed to all the remaining domesticated *V. vinifera* cultivars, for model training. Group labels were encoded as + 1, − 1, and the signs of the dependent-variable components of the partial least squares fitted models were used as decision rules for classification. Model accuracy, R^2^ and Q^2^ metrics were estimated by sevenfold stratified cross-validation. For each model, a permutation test was carried out to assess its significance, by sampling 1000 label permutations. All analysis were carried out using the package *metabolinks* (https://github.com/aeferreira/metabolinks), which uses packages scipy^60^ and scikit-learn^61^.

### Univariate statistical analysis, metabolite annotation and pathway mapping

The significance of variables in data matrices for ESI^+^ and ESI^−^ was assessed by performing two-tail *t*-tests to compare variables in “resistant/partial resistant” (n = 21) and “susceptible” (n = 12) groups. *p-*values were corrected for multiple testing by the Benjamini–Hochberg procedure. An FDR-corrected *p-*value cut-off of 0.01 was used for further consideration of a variable in the analysis. Variables were then sorted according to the fold-change defined as the ratio of the averages of “resistant/partial resistant”/“susceptible”. A variation of at least |log2(FC)| ≥ 1 was required for a variable to be considered discriminatory.

For metabolite annotation, the *m/z* values of discriminatory peaks were submitted to MassTRIX 3 server^34^ (https://masstrix.org, accessed in April 2020), allowing for the presence of adducts M +H^+^, M +K^+^ and M +Na^+^ for positive scan mode and the adducts M−H^+^ and M+Cl^−^ for negative mode. A maximum *m/z* deviation of 2 ppm was accepted; “KEGG (Kyoto Encyclopaedia of Genes and Genomes)/HMDB (Human Metabolome Database)/LipidMaps without isotopes” was selected for database search; *Vitis vinifera* was selected as the organism. For compound taxonomical classification, each KEGG’s metabolite identifier obtained from the MassTRIX search was further annotated according to the relevant ontologies of KEGG’s BRITE hierarchies^62^, if any existed for the identifier. For the “lipids” ontology the LipidMaps lipid classification system^63^ was used. Discriminatory compounds were mapped into metabolic pathways using Pathview^64^ (https://pathview.uncc.edu), selecting the *Vitis vinifera* Flavonoid biosynthesis (“vvi00941”) and Flavone and Flavonol Biosynthesis (“vvi00944”) pathways. For visualization, log2(FC) values were colour coded within the boundaries of − 5 (red, abundant in the “susceptible” group) and 5 (blue, abundant in “resistant/partial resistant” group).

### Total RNA extraction and cDNA synthesis

Total RNA was extracted from the leaves of the different *Vitis* samples using the Spectrum Plant Total RNA Kit (Sigma-Aldrich, USA), according to the manufacturer's instructions. Residual genomic DNA (gDNA) contamination was removed with On-Column DNase Digestion I Set (Sigma-Aldrich, USA), following the manufacturer's instructions. After extraction, all RNA samples were quantified, and the purity determined with the absorbance ratios at 260/280 and 260/230 nm using a NanoDrop-1000 spectrophotometer (Thermo Scientific). RNA integrity was verified by agarose gel electrophoresis. To confirm the absence of contaminating gDNA, a qPCR analysis of a target on the crude of total RNA^65,66^ was performed using *EF1α* as target. Complementary DNA (cDNA) was synthesized from 2.5 µg of total RNA using RevertAid H Minus Reverse Transcriptase (Fermentas, Ontario, Canada) anchored with Oligo(dT)_23_ primer (Sigma-Aldrich, USA), as previously described^36^. For gene expression analysis, ‘Cabernet sauvignon’ was not included in the dataset due to the lack of sufficient plant material from the same collection used for metabolomics studies.

### Reference genes selection and expression analysis

Ten candidate genes were selected based on their previous description as good qPCR reference genes for *Arabidopsis thaliana*^35^ and grapevine^36,37,67^ (Table 2). Nine of the selected genes were previously described as reference genes for grapevine: *60S ribosomal protein L18* (*60S*), *small nuclear ribonucleoprotein SmD3* [currently annotated as *Tetratricopeptide repeat protein 7B* (*TPR7B*), *elongation factor 1-alpha* (*EF1α*), *ubiquitin-conjugating enzyme* (*UBQ*), *SAND family protein* (*SAND*), *Actin* (*ACT*), *glyceraldehyde-3-phosphate dehydrogenase* (*GAPDH*), *alpha-tubulin 3-chain* (*α-TUB*) and *beta-tubulin 1-chain* (*β-TUB*)^67–71^. The other gene was retrieved from NCBI (https://www.ncbi.nlm.nih.gov/) as being homologous to *Arabidopsis adaptor protein-2 MU-adaptin* (*AP2M*).

qPCR analysis was carried out in a StepOne Real-Time PCR system (Applied Biosystems, Sourceforge, USA), using Maxima SYBR Green qPCR Master Mix (2×) kit (Fermentas, Ontario, Canada), following supplier’s instructions. Thermal cycling analysis of all genes was performed under the following conditions: initial denaturation step at 95 °C for 10 min; followed by 40 cycles of denaturation at 95 °C for 15 s plus annealing for 30 s (annealing temperatures for each primer pair were indicated at Table 4). Each set of reactions included a negative control without cDNA template. Non-specific PCR products were analysed by melting curves (see Supplementary Figure F2 online [a–j]). Three biological replicates and two technical replicates were used for each sample. To assess the amplification efficiency of each reference/candidate gene, a pool of all cDNA samples was diluted (1:4) and used to generate a five-point standard curve based on a tenfold dilution series.

### Determination of reference gene stability

To evaluate reference gene stability, all *Vitis* genotypes were analysed together and the three publicly available software tools GeNorm v. 3.5^65^, NormFinder^72^ and the BestKeeper tool^73^ were used.

GeNorm is based on the pairwise variation of a single reference candidate gene relative to all other genes. GeNorm algorithm calculates a gene expression stability measure (M value) for each gene, based on the average pairwise expression ratio between a gene and each of the other genes being compared in the analysis. Accordingly, a gene displaying a low M value presents a low variance in its expression. NormFinder is based on a variance estimation approach, which calculates an expression stability value (SV) for each gene analysed. It enables estimation of the overall variation of the reference genes, considering intra and intergroup variations of the sample set. According to this algorithm, genes with lowest SV will be top ranked^72^. The BestKeeper tool calculates standard deviation (SD) based on quantification cycle (Cq) values of all candidate reference genes^73^. Moreover, BestKeeper compares each reference gene to the BestKeeper Index (BKI) and calculate a Pearson correlation coefficient (r). Higher r values suggest more stable expression. Genes with SD less than 1 and with the highest coefficient of correlation have the highest stability. A comprehensive ranking, was established by RefFinder, a tool that integrates GeNorm, Normfinder, BestKeeper, and the comparative ΔCt method, based on the rankings from each program, allows the assignment of an appropriate weight to an individual gene and calculates the geometric mean of their weights for the overall final ranking.

A comprehensive ranking of the candidate reference genes was established by calculating the arithmetic mean of the ranking in each algorithm used, as reported previously^32^. Each gene was ranked from 1 (most stable) to 11 (least stable). The definition of the optimal number of genes required for normalization was achieved by GeNorm pairwise variation analysis^74^. Additionally, RefFinder was used as a verification tool of our results^75^ (https://www.heartcure.com.au/reffinder/).

### Selection and expression analysis of genes of interest

Genes encoding for enzymes involved in biosynthetic or catabolic reactions of the discriminatory metabolites were selected based on the fold-change of discriminatory compounds and pathway mapping.

A total of 7 genes were selected for expression analysis, coding for the following enzymes: caffeic acid O-methyltransferase (*COMT*), leucoanthocyanidin reductase 2 (*LAR2*), anthocyanidin reductase (*ANR*); fatty acyl-ACP thioesterase B (*FatB*), myo-inositol monophosphatase (*IMPL1*), flavonoid 3′,5′-hydroxylase (*F3′5′H*), and UDP-glucose:flavonoid 3-O-glucosyltransferase (*UFGT*). The selection of the genes followed the criteria of the genes being functionally described as being involved in the biosynthesis/catalysis of the compounds.

The sequences for the genes coding for the enzymes involved in catechin and epicatechin synthesis used in this study were previously described in *Vitis*^76,77^ . The remaining genes were selected by comparison of *Arabidopsis thaliana* homologue genes in the *Vitis vinifera* genome coding genes using the Basic Local Alignment Search Tool (BLAST, https://blast.ncbi.nlm.nih.gov/Blast.cgi). When gene families existed for the selected genes, the choice of the gene was made based on information on the literature regarding its involvement in plant resistance/defence.

Genes of interest selected for gene expression analysis were presented in Table 4. Non-specific PCR products were also analysed by melting curves (see Supplementary Figure F2 online [k-q]). For each gene, both standard curve efficiency and SD were calculated by the Hellemans et al. equations^78^ (Table 4).

After qPCR analysis, the quantification cycle (Cq) values of the genes of interest in all *Vitis* samples, were extracted and normalized by the geometric mean of the Cqs of *UBQ*, *SAND* and *EF1α*, described in this work as the most stable genes for sample normalization. The ability for each possible gene to discriminate between resistant/partial resistant and susceptible cultivars was assessed by testing the homocedasticity of groups with Bartlett’s test and by assessing significance of the differences between groups with a Wilcox-Mann–Whitney’s U test. All *p-*values were adjusted for false discovery rate using the Benjamini–Hochberg procedure. Results yielding an adjusted *p-*value ≤ 0.05 were considered statistically significant. Bartlett’s and   Wilcoxon–Mann–Whitney tests were performed in R^79^, using the ‘bartlett.test’, ‘wilcox.test’ and ‘p.adjust’ functions, respectively.

## Supplementary information

Supplementary Information 1.

Supplementary Figure S1.

Supplementary Figure S2.

Supplementary Table S1.

Supplementary Table S2.

## Data Availability

The metabolomics data that support the findings of this study are available in figshare data repository with the identifier https://doi.org/10.6084/m9.figshare.12357314 (https://doi.org/10.6084/m9.figshare.12357314)^80^.
