# Supplementary material for: Integrating metabolomics and targeted gene expression to uncover potential biomarkers of fungal/oomycetes-associated disease susceptibility in grapevine
Source: Sci Rep. 2020 Sep 24;10:15688. doi: 10.1038/s41598-020-72781-2 (PMC7515887; doi:10.1038/s41598-020-72781-2)
Supplement: Supplementary file 1 — Supplementary Information 1. [file 41598_2020_72781_MOESM1_ESM.docx]

**Supplementary Material**

**Supplementary Table S1 -**

Supplementary Table S1 - Different accumulated metabolites in the resistant/partial resistant and susceptible groups obtained in positive (ESI+) and negative (ESI-) ion analysis modes. Raw mass is the mass detected by FT-ICR-MS; the neutral corrected mass (without adduct), the mass in KEGG database (with adduct) and the deviation (in ppm) are indicated. The different putative metabolites, as well as their mass in KEGG, deviation, ID and formula, are separated by a #. FDR corrected p-values (≤ 0.01) and log2(FC) are indicated (log2(FC) represents the fold change between resistant and partial resistant /susceptible). The chemical formula of the compounds and the putative identification are also indicated. Compound taxonomy using KEGG BRITE or LipidMaps is presented in the second tab.

**Supplementary Table S2** **-** Gene expression analysis in the resistant/partial resistant and susceptible genotypes. Gene names' abbreviations are indicated (full gene names are indicated in Table 2). Results for Wilcox-Mann-Withney and Bartlett's tests are shown (*p*-value and adjusted *p*-value).

**Supplementary Figure F1 -** Model diagnostics showing fitting (R^2^) and prediction ability (Q^2^) metrics as a function of the number of components for the orthogonal partial least squares discriminant analysis (OPLS-DA) models for the classification into resistant/partial resistant and susceptible groups using of untargeted metabolomics data obtained in positive (c) and negative (d) ion modes. Q^2^ is calculated from stratified 7-fold cross-validation.

**Supplementary Figure F2 -** Melting curves of reference (a-j) and target (k-q) genes. a) *60S*; b) *TTC7B*; c) *EF1α*; d) *UBQ*; e) *SAND*; f) *GADPH*; g) *Actin*; h) *αTUB*; i) *AP2M*; j) *βTUB*; k) *FatB*; l) *COMT*; m) *ANR*; n) *LAR2*; o) UFGT; p) *F3’5’H*; q) *IMPL1*.
