# Supplementary figures and images for: Integrating metabolomics and targeted gene expression to uncover potential biomarkers of fungal/oomycetes-associated disease susceptibility in grapevine
Source: Sci Rep. 2020 Sep 24;10:15688. doi: 10.1038/s41598-020-72781-2 (PMC7515887; doi:10.1038/s41598-020-72781-2)

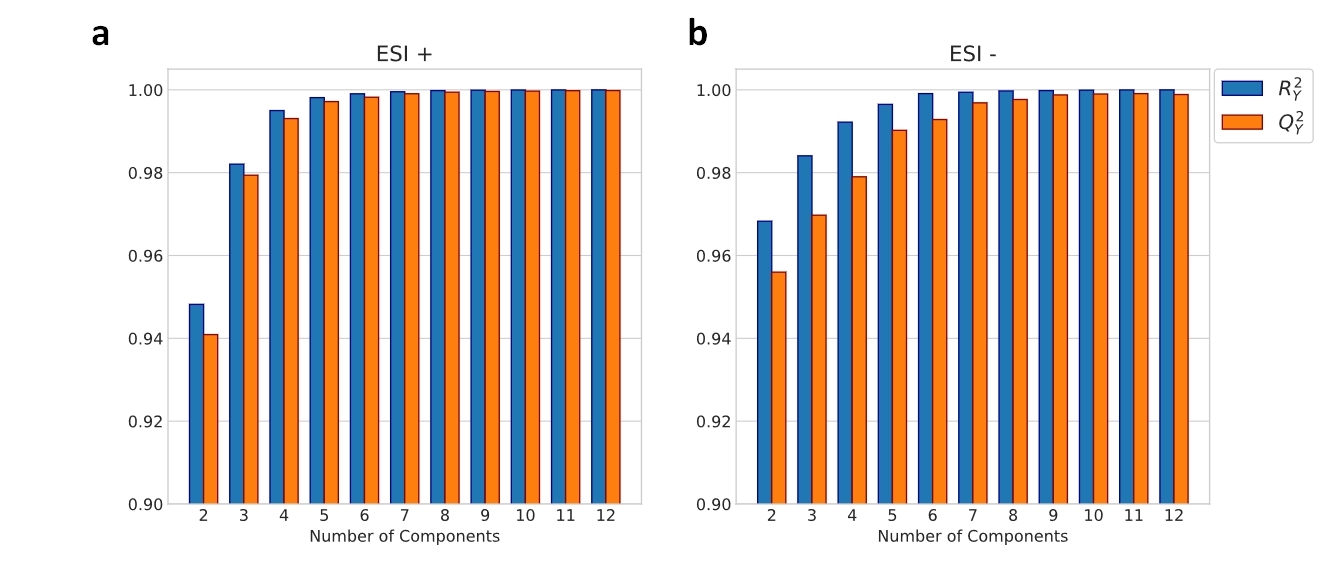

Supplement: Supplementary file 2 — Supplementary Figure S1. [file 41598_2020_72781_MOESM2_ESM.jpg]

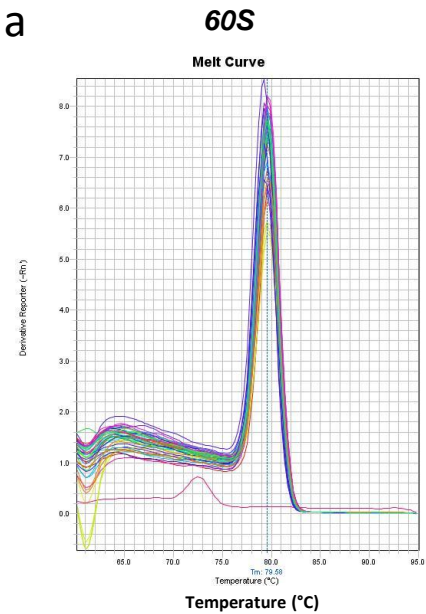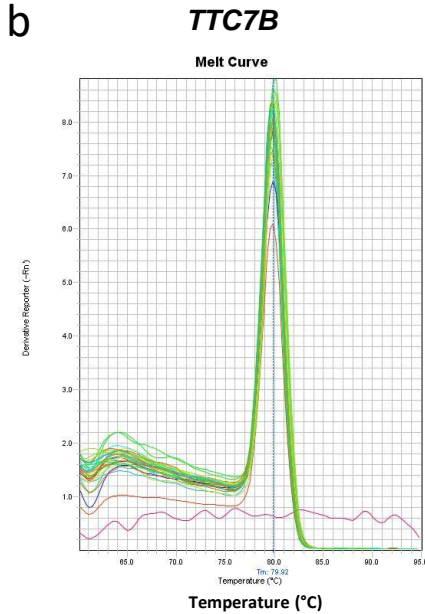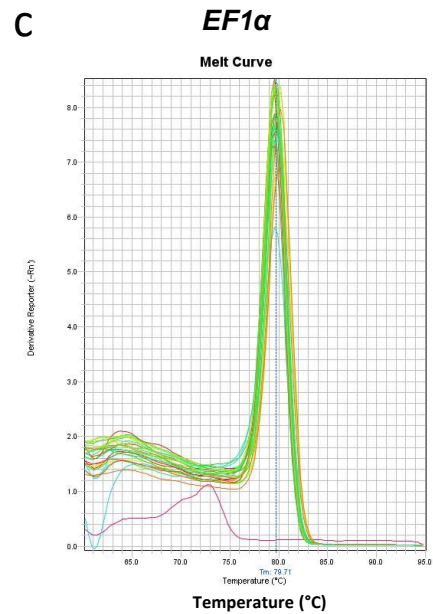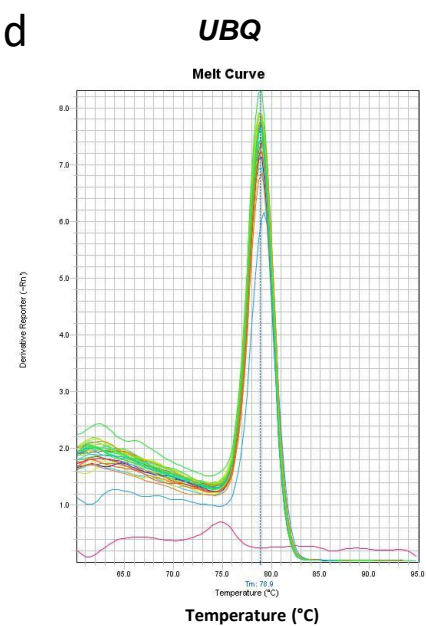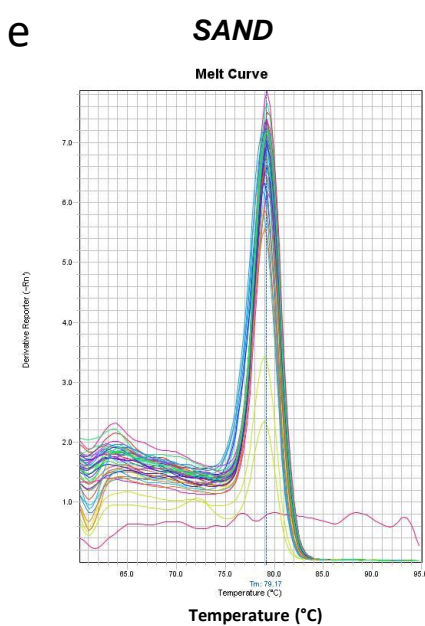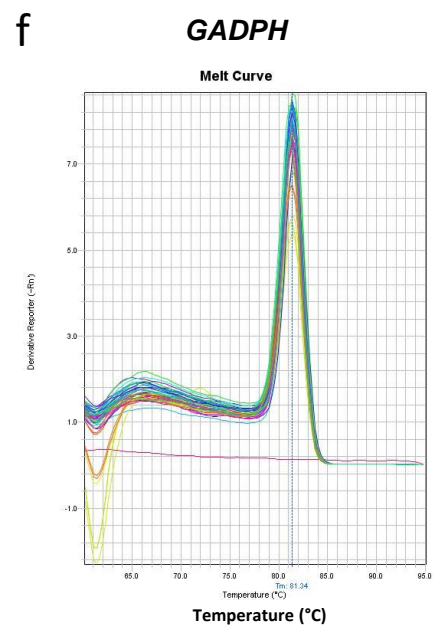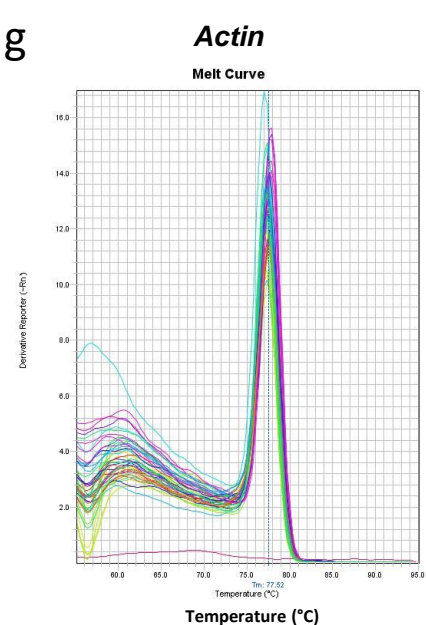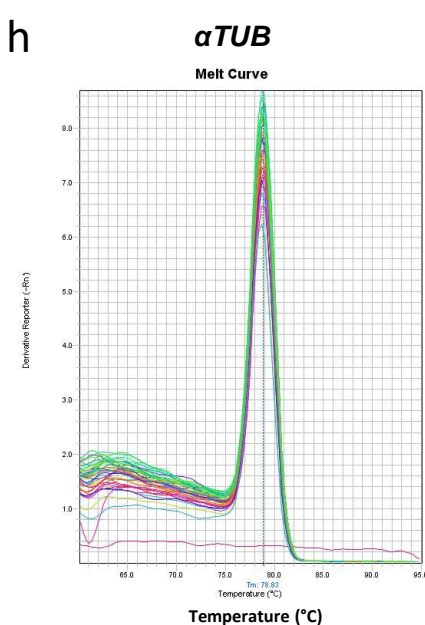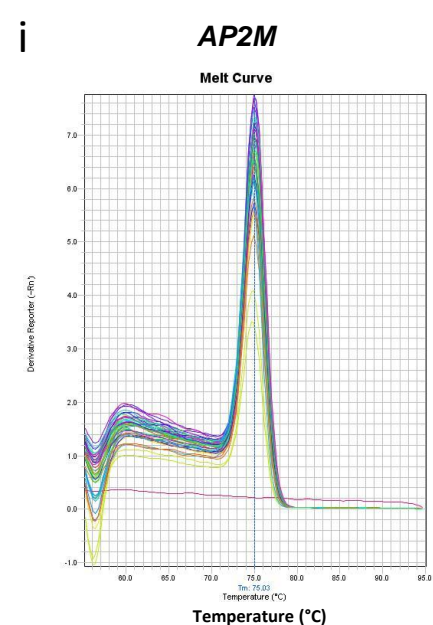

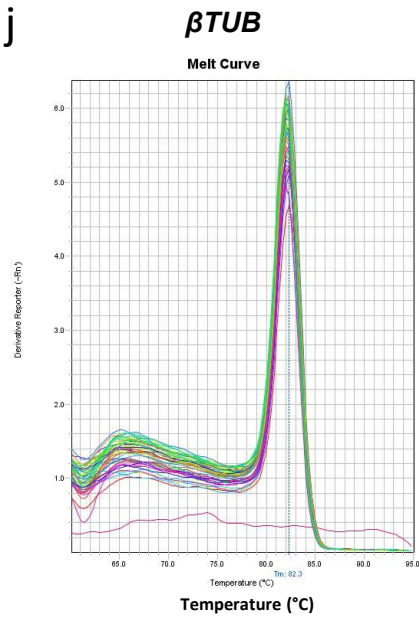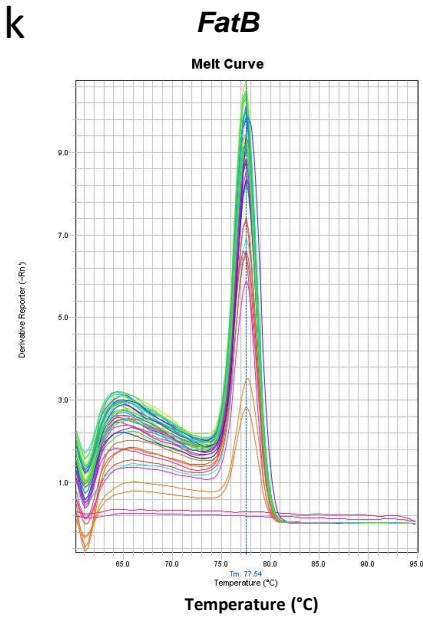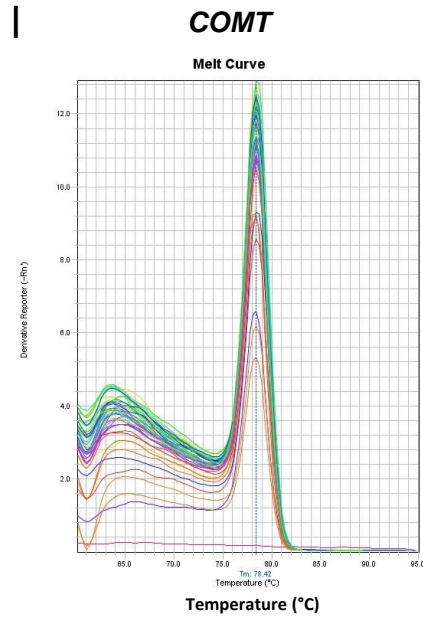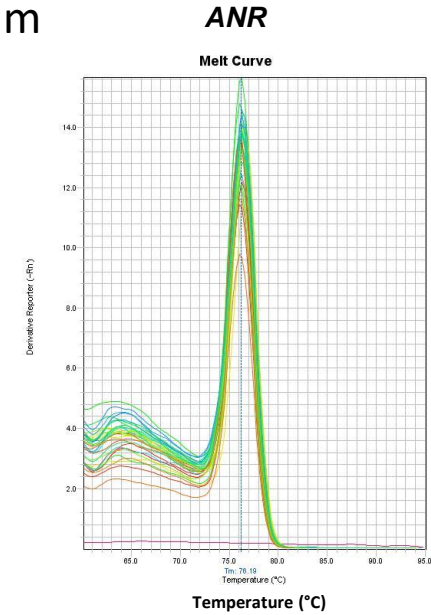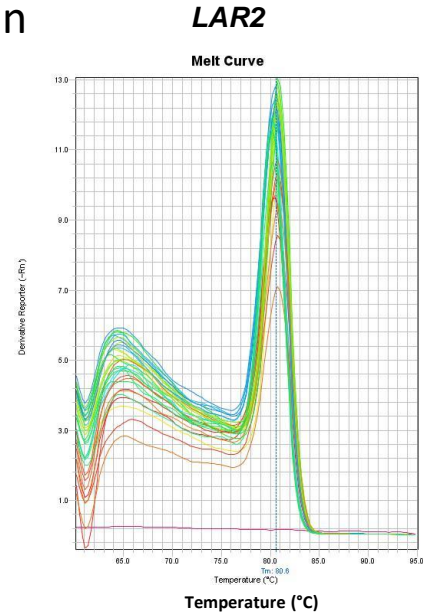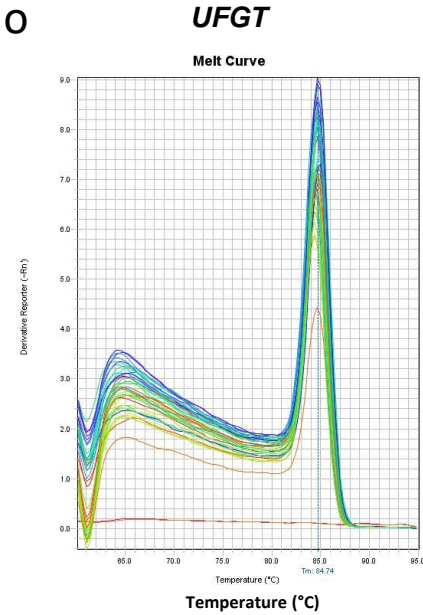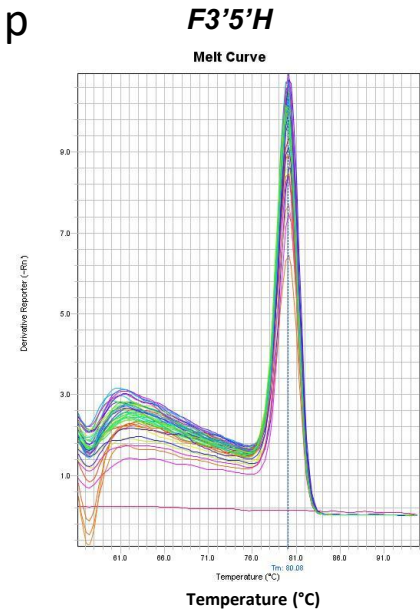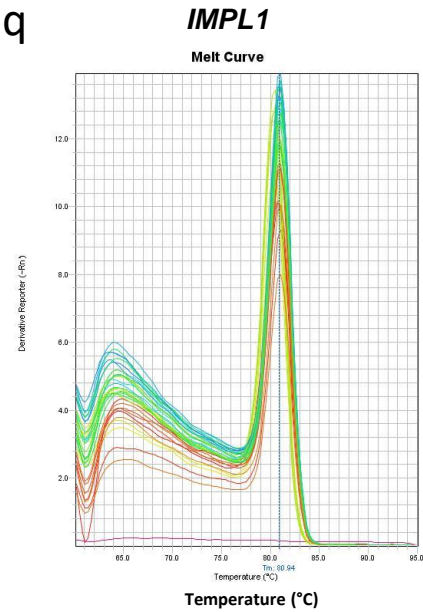

Supplement: Supplementary file 3 — Supplementary Figure S2. [file 41598_2020_72781_MOESM3_ESM.pdf]
